# Cysteinyl leukotriene E_4_ activates human group 2 innate lymphoid cells and enhances the effect of prostaglandin D_2_ and epithelial cytokines

**DOI:** 10.1016/j.jaci.2016.12.958

**Published:** 2017-10

**Authors:** Maryam Salimi, Linda Stöger, Wei Liu, Simei Go, Ian Pavord, Paul Klenerman, Graham Ogg, Luzheng Xue

**Affiliations:** a MRC Human Immunology Unit, Weatherall Institute of Molecular Medicine, NIHR Biomedical Research Centre, John Radcliffe Hospital, University of Oxford, Oxford, United Kingdom; b Respiratory Medicine Unit, Nuffield Department of Medicine, University of Oxford, Oxford, United Kingdom; c Oxford NIHR Biomedical Research Centre, Core Translational Immunology Laboratory, Oxford, United Kingdom; d Translational Gastroenterology Unit, Nuffield Department of Medicine, John Radcliffe Hospital, University of Oxford, Oxford, United Kingdom

**Keywords:** Group 2 innate lymphoid cell, leukotriene E_4_, prostaglandin D_2_, IL-25, IL-33, IL-2, thymic stromal lymphopoietin, atopic dermatitis, CRTH2, Chemoattractant receptor-homologous molecule expressed on T_H_2 cells, CSF1, Macrophage colony-stimulating factor, cysLT, Cysteinyl leukotriene, CysLT_1_, Cysteinyl leukotriene receptor 1, CysLT_2_, Cysteinyl leukotriene receptor 2, ILC2, Group 2 innate lymphoid cell, LT, Leukotriene, PGD_2_, Prostaglandin D_2_, TCR, T-cell receptor, TSLP, Thymic stromal lymphopoietin

## Abstract

**Background:**

Group 2 innate lymphoid cells (ILC2s) are a potential innate source of type 2 cytokines in the pathogenesis of allergic conditions. Epithelial cytokines (IL-33, IL-25, and thymic stromal lymphopoietin [TSLP]) and mast cell mediators (prostaglandin D_2_ [PGD_2_]) are critical activators of ILC2s. Cysteinyl leukotrienes (cysLTs), including leukotriene (LT) C_4_, LTD_4_, and LTE_4_, are metabolites of arachidonic acid and mediate inflammatory responses. Their role in human ILC2s is still poorly understood.

**Objectives:**

We sought to determine the role of cysLTs and their relationship with other ILC2 stimulators in the activation of human ILC2s.

**Methods:**

For *ex vivo* studies, fresh blood from patients with atopic dermatitis and healthy control subjects was analyzed with flow cytometry. For *in vitro* studies, ILC2s were isolated and cultured. The effects of cysLTs, PGD_2_, IL-33, IL-25, TSLP, and IL-2 alone or in combination on ILC2s were defined by using chemotaxis, apoptosis, ELISA, Luminex, quantitative RT-PCR, and flow cytometric assays. The effect of endogenous cysLTs was assessed by using human mast cell supernatants.

**Results:**

Human ILC2s expressed the LT receptor CysLT_1_, levels of which were increased in atopic subjects. CysLTs, particularly LTE_4_, induced migration, reduced apoptosis, and promoted cytokine production in human ILC2s *in vitro*. LTE_4_ enhanced the effect of PGD_2_, IL-25, IL-33, and TSLP, resulting in increased production of type 2 and other proinflammatory cytokines. The effect of LTE_4_ was inhibited by montelukast, a CysLT_1_ antagonist. Interestingly, addition of IL-2 to LTE_4_ and epithelial cytokines significantly amplified ILC2 activation and upregulated expression of the receptors for IL-33 and IL-25.

**Conclusion:**

CysLTs, particularly LTE_4_, are important contributors to the triggering of human ILC2s in inflammatory responses, particularly when combined with other ILC2 activators.

Group 2 innate lymphoid cells (ILC2s) are recognized as an innate source of type 2 cytokines in the pathogenesis of allergic conditions, such as asthma and atopic dermatitis.^1^, ^2^ ILC2s are known to be lymphoid effector cells that do not express rearranged antigen-specific receptors but express CD45, IL-7 receptor α, and chemoattractant receptor-homologous molecule expressed on T_H_2 cells (CRTH2) while lacking lineage markers, including CD3, T-cell receptor (TCR) αβ, TCRγδ, CD14, CD19, CD56, CD11b, and CD11c. Although they can be found in various anatomic locations, their enrichment in mucosal surfaces of the lung, gut, and skin implies a local immunologic role.^2^, ^3^, ^4^, ^5^ ILC2s contribute to allergic and inflammatory conditions by producing IL-4, IL-5, IL-13, and GM-CSF. In the gut ILC2s are a source of type 2 cytokines for efficient expulsion of the helminth *Nippostrongylus brasiliensis*.^1^ In the lungs they induce airway hyperresponsiveness and epithelial repair after influenza infection.^3^, ^6^

ILC2s not only respond to the epithelial cytokines IL-33, IL-25, and thymic stromal lymphopoietin (TSLP) but also respond to the mast cell lipid mediator prostaglandin D_2_ (PGD_2_).^2^, ^7^ It has also been reported that murine ILC2s respond to another group of lipid mediators, cysteinyl leukotrienes (cysLTs), to produce type 2 cytokines, although the effect of cysLTs in human ILC2s is still unclear.^8^

CysLTs, including leukotriene (LT) C_4_, LTD_4_, and LTE_4_, are formed from arachidonic acid metabolism.^9^ At the nuclear envelope, cytosolic phospholipase A_2_ liberates membrane arachidonic acid, which binds to 5-lipoxygease–activating protein. 5-Lipoxygenase catalyzes the formation of LTA_4_ by adding an oxygen moiety to arachidonic acid. Activated inflammatory cells, such as eosinophils, basophils, mast cells, and alveolar macrophages, possessing LTC_4_ synthase can synthesize LTC_4_ rapidly through conjugation of LTA_4_ with reduced glutathione levels.^10^ After extracellular export, LTC_4_ is converted to LTD_4_ and then LTE_4_ by means of sequential removal of the glutamic acid moiety, followed by the glycine moiety. LTD_4_ is the most potent airway muscle contractant with the shortest half-life (in minutes); in contrast, LTE_4_ is stable and the dominant LT detected in biological fluids.^11^ Monitoring LTE_4_ levels in urine, sputum, and exhaled air is an index of the activity of the cysLT synthesis pathway.^12^ The surge in cysLT production associates with an increase in microvascular permeability, eosinophil recruitment, mucus hypersecretion, bronchoconstriction, and cell proliferation.^13^ The role of cysLTs in the pathogenesis of allergic conditions, such as asthma, allergic rhinitis, urticaria, and other inflammatory conditions, has been well studied.^9^ We reported recently that cysLTs potentiated the proinflammatory functions of T_H_2 cells in response to PGD_2_.^14^, ^15^ Two G protein–coupled receptors for cysLTs have been characterized and designated as cysteinyl leukotriene receptor 1 (CysLT_1_), with high binding affinity for LTD_4_, and cysteinyl leukotriene receptor 2 (CysLT_2_), with similar affinity for LTD_4_ and LTC_4_, but both receptors have low affinity for LTE_4_.^16^, ^17^ Other potential receptors for LTE_4_ include adenosine diphosphate–reactive purinoceptor P2Y_12_, with the highest homology to CysLT_1_ (32%) and GPR99.^18^, ^19^

CysLT_1_ mediates bronchoconstriction and also a range of proinflammatory effects, including activation and migration of leukocytes.^20^, ^21^ CysLT_1_ antagonists, most notably montelukast, are used to control asthma and allergic rhinitis. Definition of the role of cysLTs and their receptors in human ILC2s will improve our understanding of the pathogenic mechanisms of ILC2-mediated allergic inflammation and indicate potential novel therapeutic strategies.

In this study we explored the effect of cysLTs and their receptors on human ILC2s, particularly when combined with other ILC2 stimulators. We investigated whether human ILC2s express functional CysLT_1_ and whether expression is increased by ILC2s from patients with atopic dermatitis. We assessed the effects of cysLTs on cytokine production, migration, and apoptosis of cells in the presence and absence of the CysLT_1_ antagonist montelukast. Finally, we assessed whether cysLTs show a synergistic effect with PGD_2_ and epithelial cytokines in activating the cells. Our study provides a broad understanding of the role of cysLTs in ILC2-mediated inflammatory responses, particularly in mixed inflammatory environments.

## Methods

### ILC2 cell preparation and culture

Human ILC2s were prepared from human blood from healthy donors and cultured by using a modified method, as described previously.^2^ Briefly, PBMCs were isolated by using Lymphoprep gradients (Axis-Shield UK, Dundee, United Kingdom). CD3^+^ T cells were predepleted with CD3 microbeads, and the remaining cells were labeled with an antibody mixture. Lineage (CD3, CD14, CD19, CD56, CD11b, CD11c, TCRαβ, TCRγδ, FcεRI, and CD123)–negative, CD45^high^, CD127^+^, and CRTH2^+^ cells were sorted on a FACSAria III cell sorter (BD Biosciences, San Jose, Calif) and cultured for 5 to 6 weeks in RPMI 1640 containing 100 IU/mL IL-2, 10% heat-inactivated human serum, 2 mmol/L l-glutamine, 100 IU/mL penicillin, and 100 μg/mL streptomycin in the presence of gamma-irradiated PBMCs (from 3 healthy volunteers). Half of the medium was refreshed every 2 to 3 days. The cells were changed to fresh medium without IL-2 before treatment. Most ILC2s used in the study were derived from healthy donors, except where indicated specifically.

Adult (30-88 years) patients with atopic dermatitis received a diagnosis based on the United Kingdom refinements of the Hanifin and Rajka diagnostic criteria, and the disease severity score was defined by using SCORAD. None of the patients were receiving systemic therapy at the time of the sample acquisition. Use of human tissue samples was approved ethically by the Oxford Clinical Research Committee.

### Human mast cell culture and activation

Human mast cells were cultured from CD34^+^ progenitor cells and treated with human IgE and goat anti-human IgE in the presence or absence of MK886, as described previously.^14^ Cell supernatants were collected and LTE_4_ levels were measured with an ELISA, or the supernatants were stored at −80°C until used as mast cell supernatants for the treatment of ILC2s.

### Chemotaxis assays

Cell migration assays were conducted, as described previously.^7^ Briefly, ILC2s (approximately 5 × 10^4^ cells/well) and treatment reagents were loaded into the upper and lower chambers, respectively, in a 5-μm pore–sized ChemoTx plate (Neuro Probe, Gaithersburg, Md). After incubation for 1 hour, migrated cells in the lower chambers were treated with a Cell Titer-Glo Luminescent Cell Viability Assay kit (Promega, Madison, Wis) and quantified by using a FLUOstar OPTIMA luminescence plate reader (BMG LabTech, Cary, NC).

### Apoptosis assay

ILC2s (approximately 5 × 10^5^ cells per condition) were harvested after different treatments and transferred to annexin-binding buffer, followed by incubation with phycoerythrin–Annexin V/propidium iodide at room temperature, according to the manufacturer's instructions (Invitrogen, Carlsbad, Calif). The stained cells were analyzed with an LSR Fortessa flow cytometer (BD Biosciences).

### Multiplex bead array

After treatment for 4 hours, concentrations of selected cytokines in the supernatants of ILC2 (approximately 6 × 10^5^ cells/well) cultures were measured with a Human Premixed Multi-Analyte Kit (R&D Systems, Minneapolis, Minn) with magnetic beads, according to the manufacturer's instruction. Results were obtained with a Bio-Plex 200 System (Bio-Rad Laboratories, Hercules, Calif).

### Quantitative RT-PCR

Quantitative RT-PCR was conducted, as described previously.^7^ Primers and probes (Roche, Mannheim, Germany) are listed in Table E1 in this article's Online Repository at www.jacionline.org.

### ELISA

Concentrations of cytokines in the supernatants of ILC2 cultures (approximately 6 × 10^5^ cells/well) were assayed with ELISA kits (R&D Systems). LTE_4_ levels in supernatants of mast cells were assayed with an LTE_4_ enzyme immunoassay kit (Cayman Chemical, Ann Arbor, Mich). Results were measured with a FLUOstar OPTIMA luminescence plate reader (BMG LabTech).

### Flow cytometric analysis

PBMCs from patients or ILC2s from cultures were fluorescently labeled with antibodies and acquired by using Summit and FACSDiva software on a CyAn Flow Cytometer (Beckman Coulter, Fullerton, Calif) and LSR Fortessa, respectively. The flow cytometric data were analyzed by using FlowJo software (TreeStar, Ashland, Ore).

### Statistics

Data were analyzed by using 1-way ANOVA, followed by the Newman-Keuls test or *t* test. *P* values of less than .05 were considered statistically significant. Data are presented as means ± SEMs.

## Results

### Human ILC2s express functional CysLT_1_, with higher expression in atopic subjects

**Fig 1:**
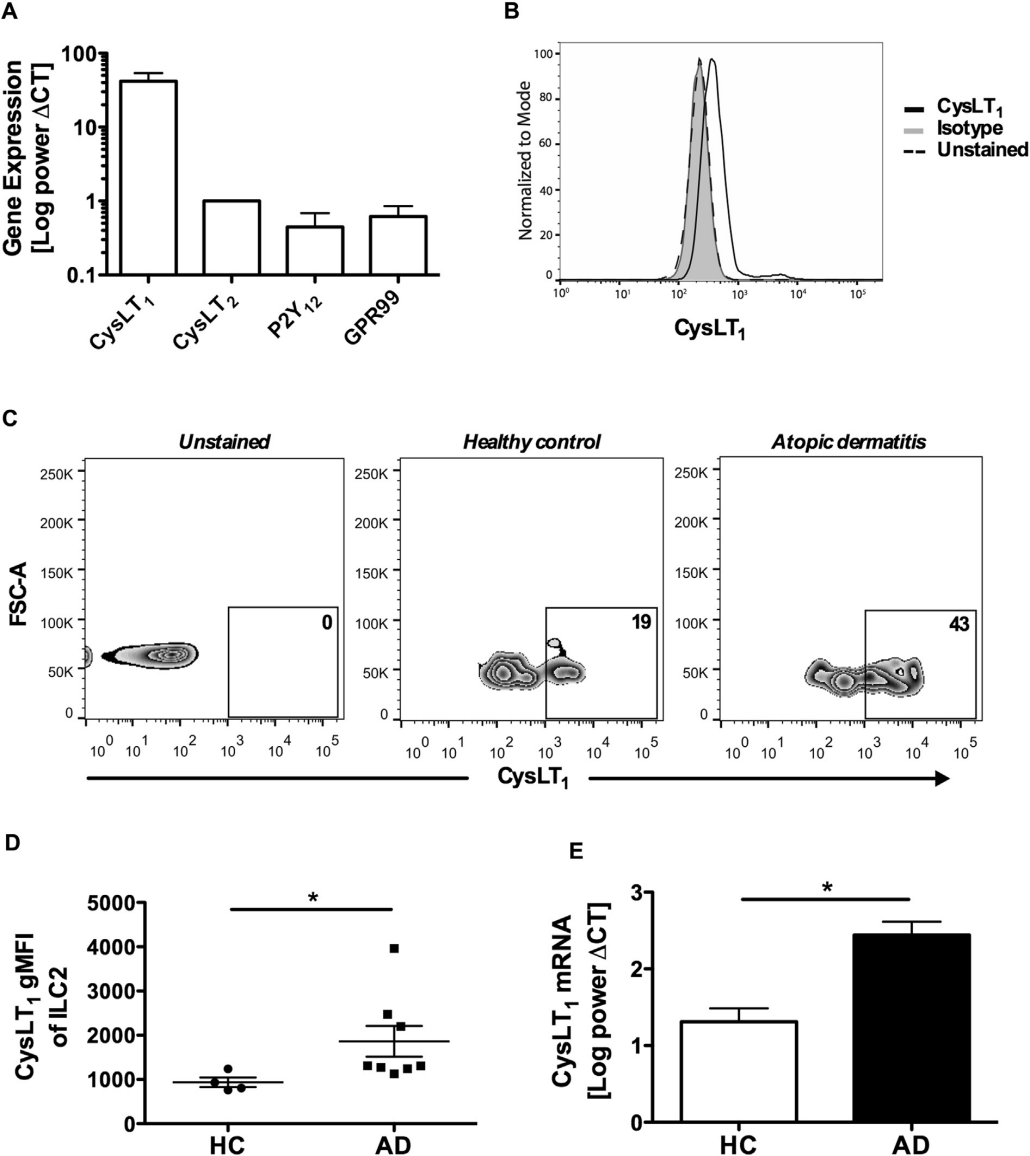
Increased expression of CysLT_1_ by ILC2s in patients with atopic dermatitis *(AD).***A,** mRNA for cysLT receptors in cultured ILC2s with quantitative RT-PCR. **B,** Expression of CysLT_1_ with flow cytometry. **C** and **D,** CysLT_1_^+^ ILC2s detected in PBMCs from healthy subjects and patients with AD (Fig 1, *C*) and cohort mean fluorescence intensity CysLT_1_ (Fig 1, *D*). *FSC*, Forward scatter. **E,** mRNA for CysLT_1_ in blood ILC2s. *P < .05 (Fig 1, *A*, n = 10; Fig 1, *D*, n = 4-8; Fig 1, *E*, n = 3).

**Fig E1:**
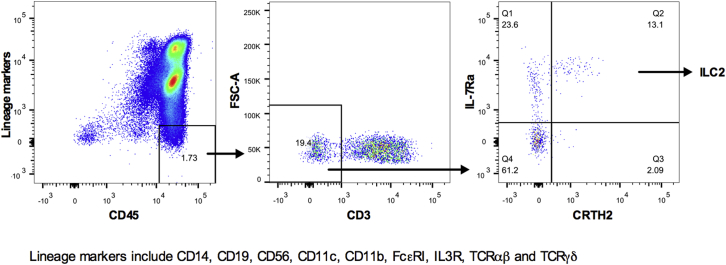
ILC2 isolation. ILC2s isolated from human blood were lineage marker negative (CD3, CD14, CD19, CD56, CD11c, CD11b, FcεRI, TCRγδ, TCRα,β and CD123), CD45^high^, IL-7 receptor α–positive, and CRTH2^+^ cells. *FSC*, Forward scatter.

**Fig E2:**
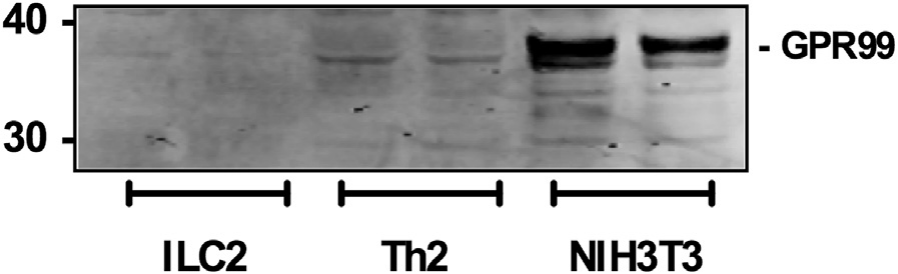
GPR99 is not expressed by human ILC2s and T_H_2 cells. The protein level of GPR99 in cell lysates was detected by using Western blotting. NIH3T3 cells were used as a positive control.

To investigate the effect of cysLTs on human ILC2s, we first examined the expression of cysLT receptors in cells *ex vivo*. Human ILC2s were prepared from human blood (gating strategy is shown in Fig E1 in this article's Online Repository at www.jacionline.org).^2^, ^7^ Transcriptional levels of the established leukotriene receptors CysLT_1_ and CysLT_2_ and the putative receptors P2Y_12_ and GPR99 in cells were measured (Fig 1, *A*). Similar to what has been previously observed in T_H_2 cells,^22^ the transcriptional level of CysLT_1_ in ILC2s was high. Compared with CysLT_1_, the level of CysLT_2_ was much lower in the cells. For P2Y_12_ and GPR99, only trace levels of mRNA (Fig 1, *A*) but not protein (see Fig E2 in this article's Online Repository at www.jacionline.org) were detected. Expression of CysLT_1_ on the cell surface was confirmed by using flow cytometry (Fig 1, *B*).

Because ILC2s play an important part in the initiation and maintenance of allergic inflammation in atopic lesions,^2^, ^5^ we further compared the level of CysLT_1_ in ILC2s isolated *ex vivo* from patients with atopic dermatitis and healthy control subjects (Fig 1, *C*, and see Table E2 in this article's Online Repository at www.jacionline.org). CysLT_1_ expression was significantly higher in cells from atopic subjects at both the protein (Fig 1, *D*) and mRNA (Fig 1, *E*) levels.

### LTs mediate migration of human ILC2s

**Fig 2:**
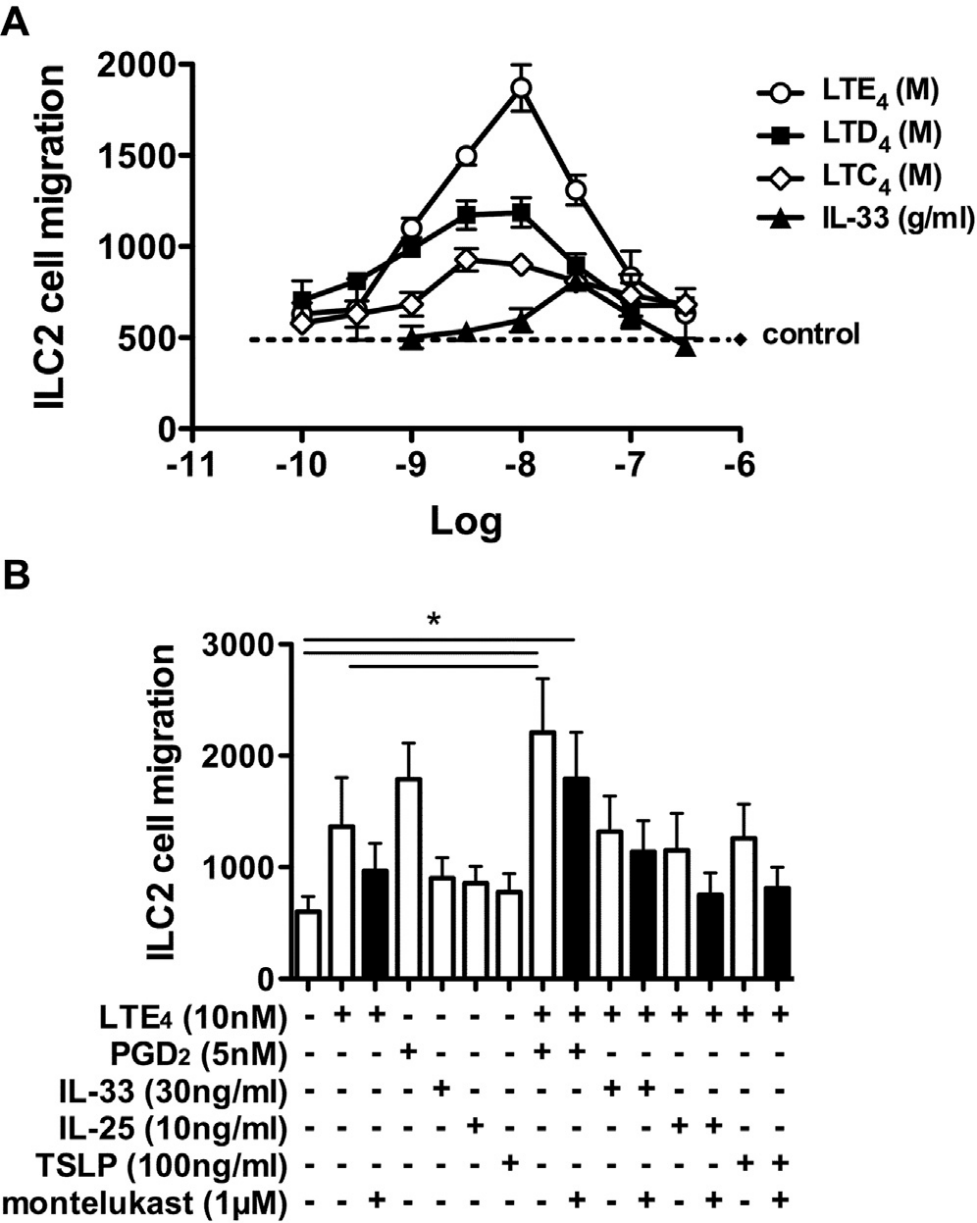
Migration of cultured ILC2s in response to cysLTs. **A,** Migration of ILC2s to cysLTs or IL-33 examined with chemotaxis assays. **B,** Migration of ILC2s in response to LTE_4_, PGD_2_, IL-33, IL-25, and TSLP alone or in combination in the presence/absence of montelukast. *P < .05. Data in Fig 2, *A*, are representative of 4 experiments (Fig 2, *B*, n = 3).

CysLTs are strong chemotactic agents for eosinophils, monocytes, T_H_2 cells, and hematopoietic progenitor cells.^15^, ^22^, ^23^, ^24^, ^25^ To determine the role of cysLTs on ILC2 migration, we tested the effect of LTC_4_, LTD_4_, and LTE_4_ using a transwell chemotaxis assay and compared them with IL-33, an established ILC2 chemokine.^2^ All 3 cysLTs induced ILC2 chemotaxis in a dose-dependent manner, peaking at about 3 nmol/L for LTC_4_ and LTD_4_ and 10 nmol/L for LTE_4_ (Fig 2, *A*). The maximum response achieved with LTE_4_ was greater than that of the other 2 cysLTs. LTs, particularly LTE_4_, showed a significantly stronger effect than IL-33 on ILC2 chemotaxis. The combination of LTE_4_ and PGD_2_ enhanced the chemotaxis, but this additive effect was not observed when LTE_4_ was combined with IL-25, IL-33, or TSLP (Fig 2, *B*). The chemotactic effect of cysLTs in ILC2s was inhibited by montelukast.

### CysLTs promote survival of human ILC2s

**Fig 3:**
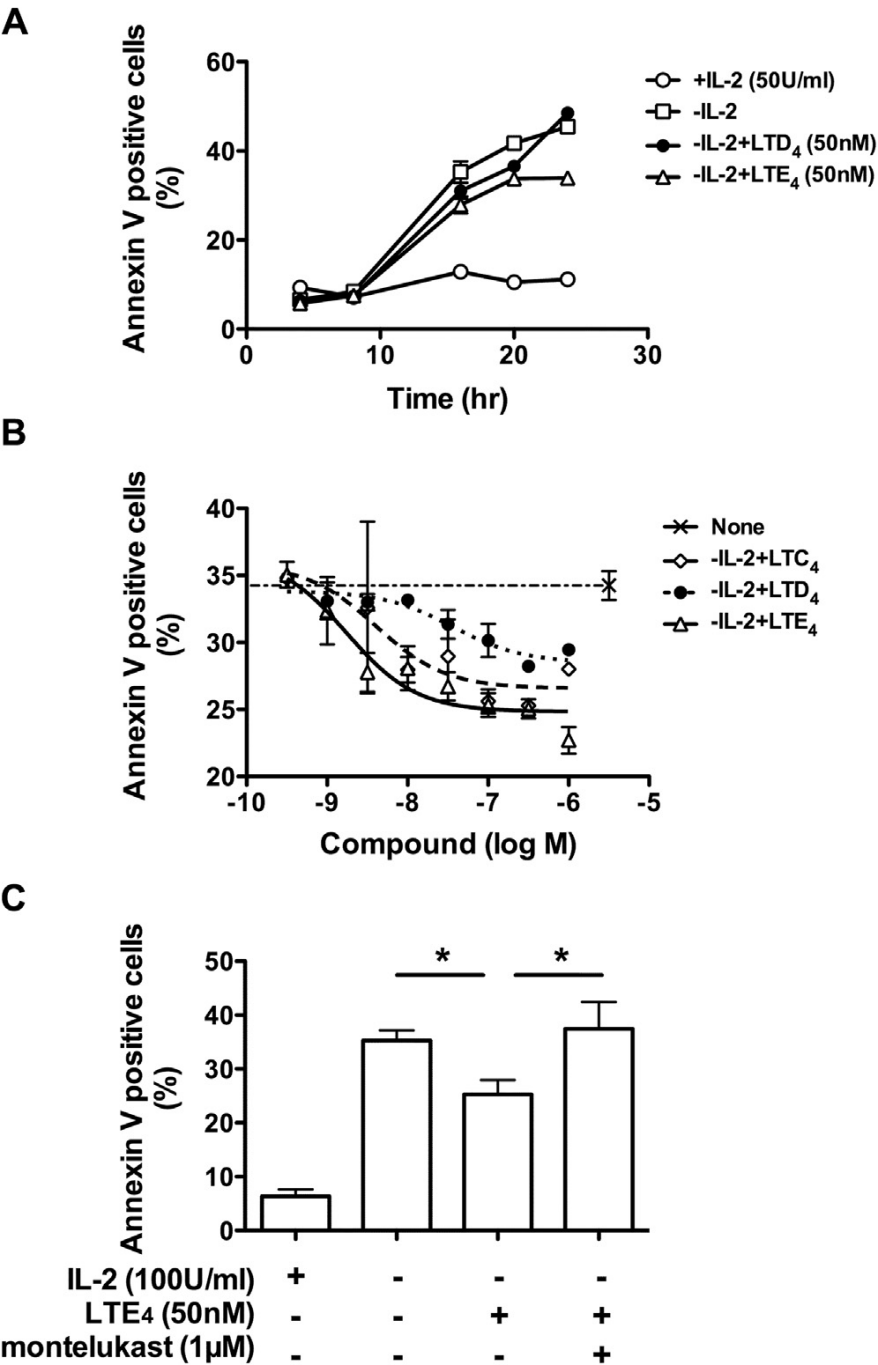
Effect of cysLTs on apoptosis of cultured ILC2s. **A,** Annexin-V^+^ cells detected by using flow cytometry after IL-2 withdrawal in the presence/absence of cysLTs. **B** and **C,** Annexin-V^+^ cells at 18 hours after IL-2 withdrawal in the presence of LTC_4_*(dashed line)*, LTD_4_*(dotted line)*, or LTE_4_ (*solid line*; Fig 3, *B*) or LTE_4_/montelukast (Fig 3, *C*). *P < .05. Data in Fig 3, *A*, are representative of 3 experiments (Fig 3, *B*, n = 4; Fig 3, *C*, n = 3).

**Fig E3:**
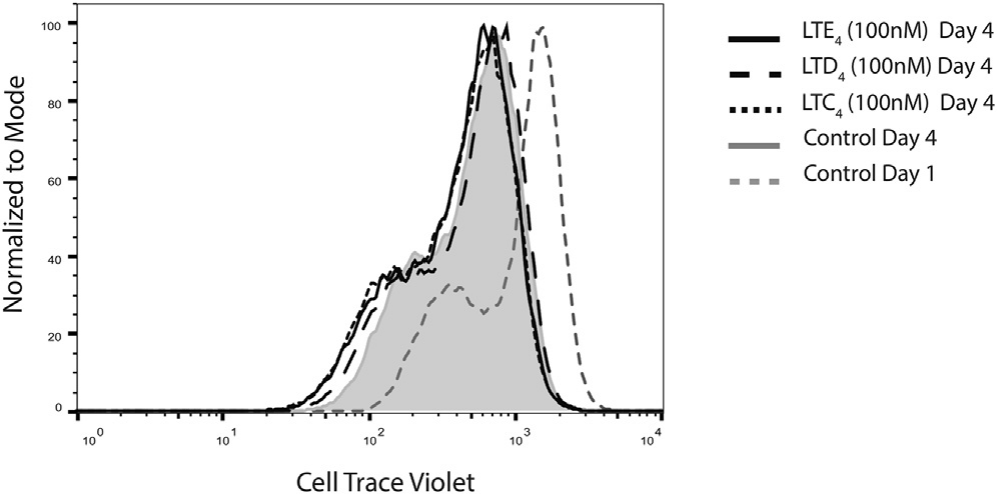
CysLTs do not affect proliferation of ILC2s in culture. Levels of Cell Trace Violet in ILC2s were measured with flow cytometry at day 4 after cell labeling with Cell Trace Violet and then incubation with 100 nmol/L LTC_4_, LTD_4_, or LTE_4_ (n = 3).

LTs contribute to eosinophil and neutrophil survival during inflammatory responses.^26^, ^27^ We next examined the effect of cysLTs on ILC2 survival using an apoptosis assay. Removal of IL-2 led to a dramatic increase in the number of Annexin V–positive ILC2s (Fig 3, *A*). The increase in Annexin V binding was reduced by LTD_4_ (50 nmol/L) or LTE_4_ (50 nmol/L) within 20 hours. However, because of the degradation of early apoptotic cells, the precise potency of cysLTs on ILC2 apoptosis beyond 24 hours could not be determined. The prosurvival effect of cysLTs on ILC2s was concentration dependent, with a half maximal inhibitory concentration at 4, 36, and 1.8 nmol/L for LTC_4_, LTD_4_, and LTE_4_, respectively (Fig 3, *B*). LTE_4_ was more potent in inhibiting ILC2 apoptosis. In contrast, no effect of cysLTs on ILC2 proliferation was detected (see Fig E3 in this article's Online Repository at www.jacionline.org). The antiapoptotic effect of cysLTs was abolished by montelukast (Fig 3, *C*).

### CysLTs induce production of type 2 cytokines by human ILC2s

**Fig 4:**
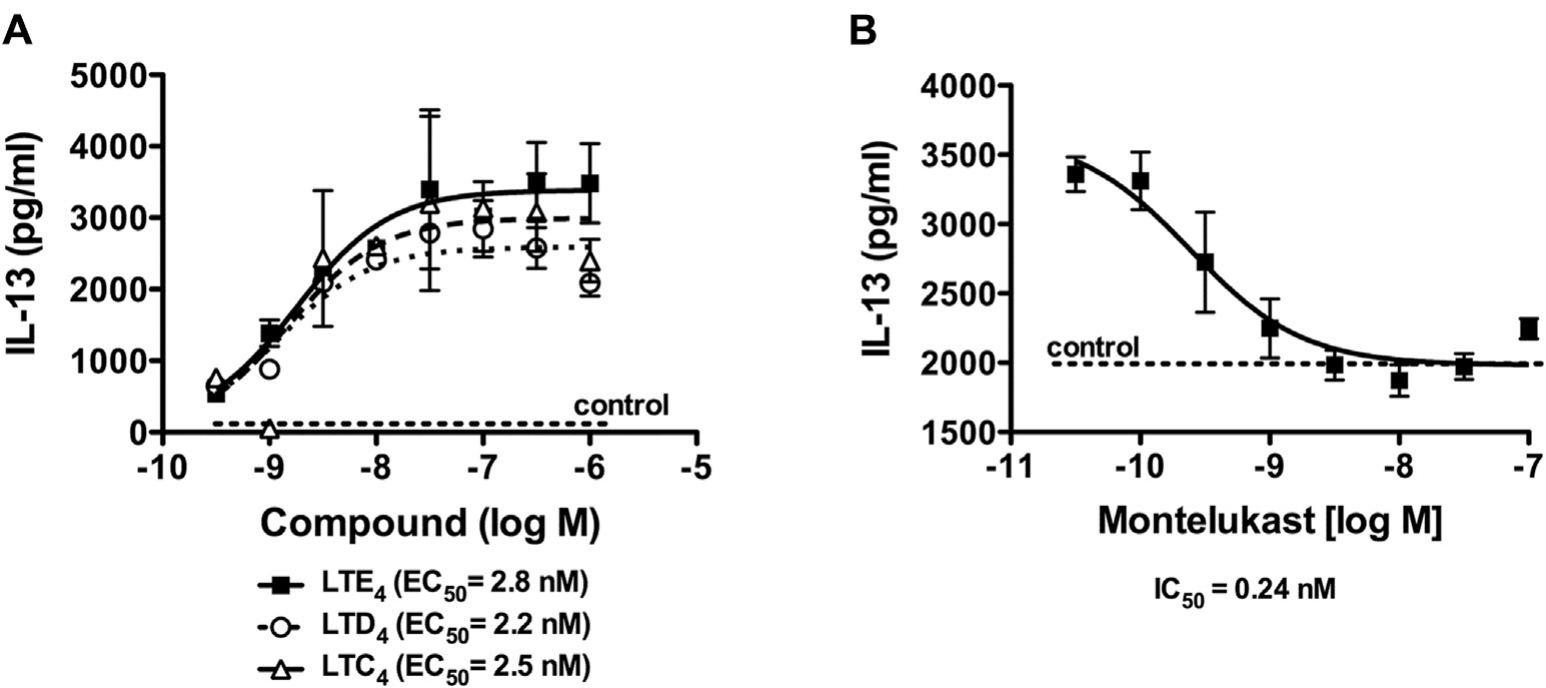
Effect of cysLTs and CysLT_1_ antagonist on IL-13 production in cultured ILC2s. **A,** IL-13 concentrations in cell supernatants measured with ELISA after treatment with LTC_4_*(dashed line)*, LTD_4_*(dotted line)*, or LTE_4_*(solid line)*. **B,** IL-13 production by ILC2s induced by 100 nmol/L LTE_4_ in the presence of montelukast. Data in Fig 4, *A*, are representative of 5 experiments (Fig 4, *B*, n = 1). *EC*_*50*_, Half maximal effective concentration; *IC*_*50*_, half maximal inhibitory concentration.

**Fig 5:**
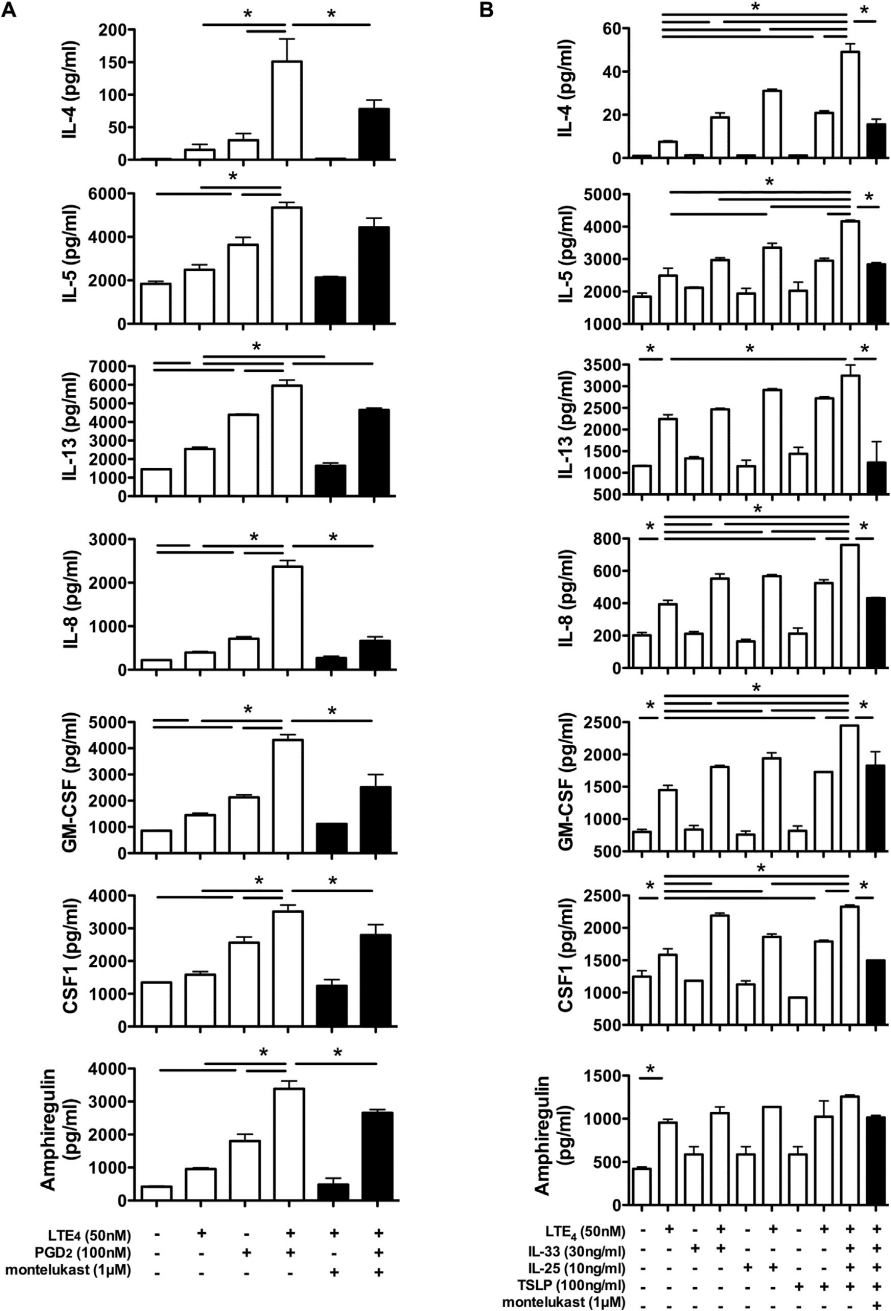
LTE_4_ enhancement of effects of PGD_2_ and epithelial cytokines on cytokine production by cultured ILC2s. Cytokine concentrations after ILC2 treatment with LTE_4_ and PGD_2_ alone or in combination **(A)** or LTE_4_, IL-33, IL-25, and TSLP alone or in combination **(B)** in the presence *(black bar)*/absence *(white bars)* of montelukast. Cytokines were measured with the multiplex bead array. *P < .05 (n = 3).

One of the important proinflammatory roles of cysLTs is enhancing type 2 cytokine production.^8^, ^14^, ^15^ To investigate this effect in human ILC2s, we incubated ILC2s with increasing concentrations of LTC_4_, LTD_4_, and LTE_4_. All LTs enhanced IL-13 production in a dose-dependent manner with a similar half maximal effective concentration: 4 ± 1.5, 4.1 ± 1.9, and 3.9 ± 1.1 nmol/L for LTC_4_, LTD_4_, and LTE_4_, respectively (Fig 4, *A*). Other type 2 cytokines (IL-4 and IL-5) were also induced by cysLTs (Fig 5, data not shown). Because LTE_4_ induced greater responses than LTC_4_ and LTD_4_ by ILC2s, we subsequently focused on LTE_4_. The effect of montelukast was examined to investigate the role of CysLT_1_ on the cytokine production induced by cysLTs. Montelukast exhibited dose-dependent inhibition, with a half maximal inhibitory concentration at 0.24 nmol/L for 100 nmol/L LTE_4_ treatment (Fig 4, *B*). Ten to 30 nmol/L was required to completely abolish the effect of LTE_4_ on cytokine production.

### LTE_4_ enhances the effect of PGD_2_ and epithelial cytokines on activation of human ILC2s

**Fig E4:**
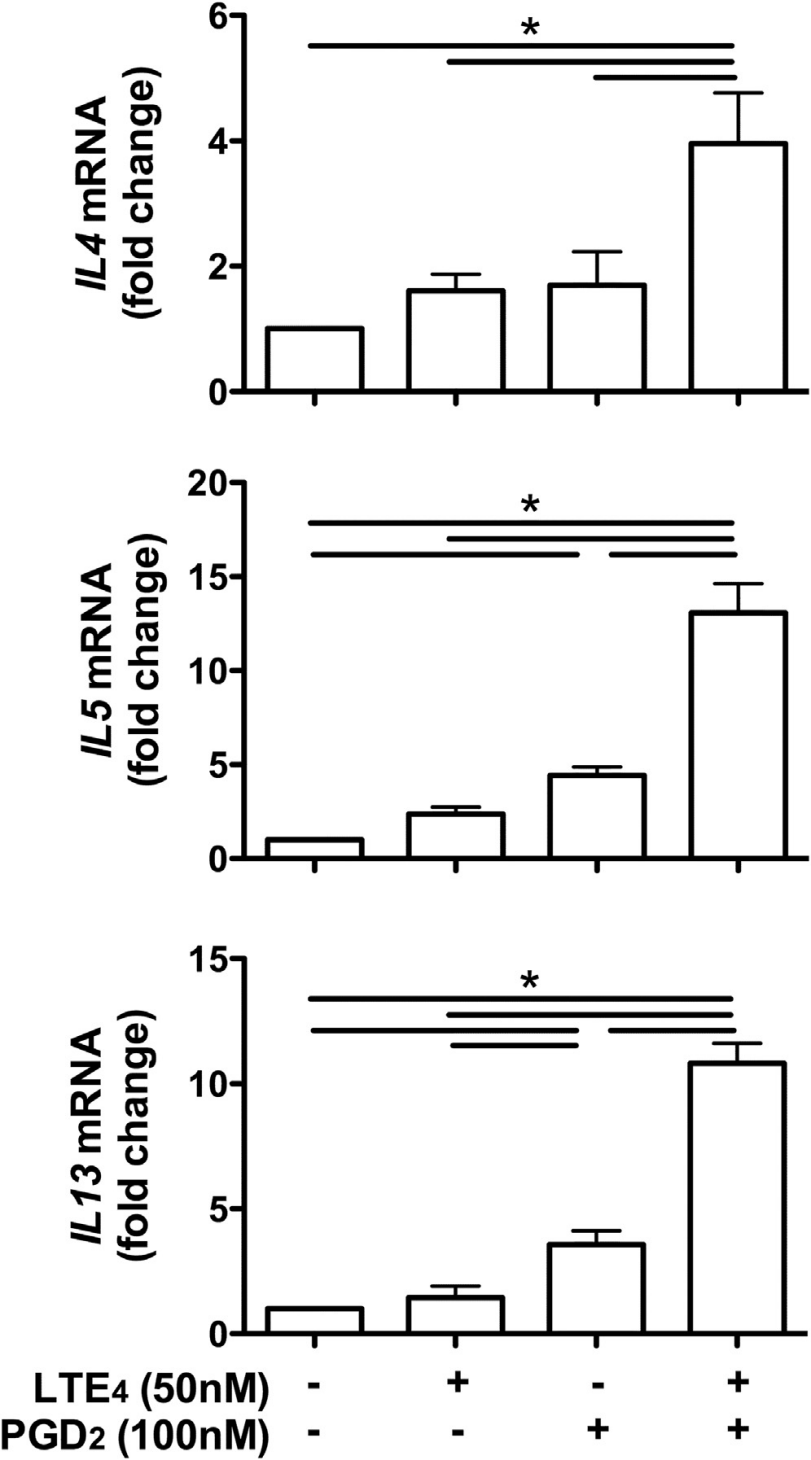
LTE_4_ synergistically enhanced gene transcription of type 2 cytokines induced by PGD_2_ in cultured human ILC2s. mRNA levels of type 2 cytokines in ILC2s after treatment with LTE_4_ and PGD_2_ alone or their combination for 4 hours were determined by using quantitative RT-PCR. *P < .05 (n = 3).

Our previous report demonstrated that PGD_2_ could stimulate the production of multiple cytokines (IL-3, IL-4, IL-5, IL-8, IL-13, GM-CSF, and macrophage colony-stimulating factor [CSF1]) by human ILC2s.^7^ The cells were treated with these lipids alone or in combination to further understand the effect of combinations of cysLTs and PGD_2_ on ILC2s. Compared with PGD_2_, LTE_4_ only weakly increased production of IL-4, IL-5, IL-8, IL-13, GM-CSF, CSF1, and amphiregulin (Fig 5, *A*). The combination of LTE_4_ and PGD_2_ exhibited synergistic (IL-4, IL-8, GM-CSF, CSF1, and amphiregulin) or additive (IL-5 and IL-13) enhancement of cytokine production. The effect associated with LTE_4_ was removed by montelukast. mRNA levels of type 2 cytokines were measured by using quantitative RT-PCR to confirm the synergistic effect of LTE_4_ and PGD_2_ at the gene level. The combination of LTE_4_ and PGD_2_ showed further enhancement in gene transcription levels compared with LTE_4_ or PGD_2_ alone (see Fig E4 in this article's Online Repository at www.jacionline.org).

**Fig E5:**
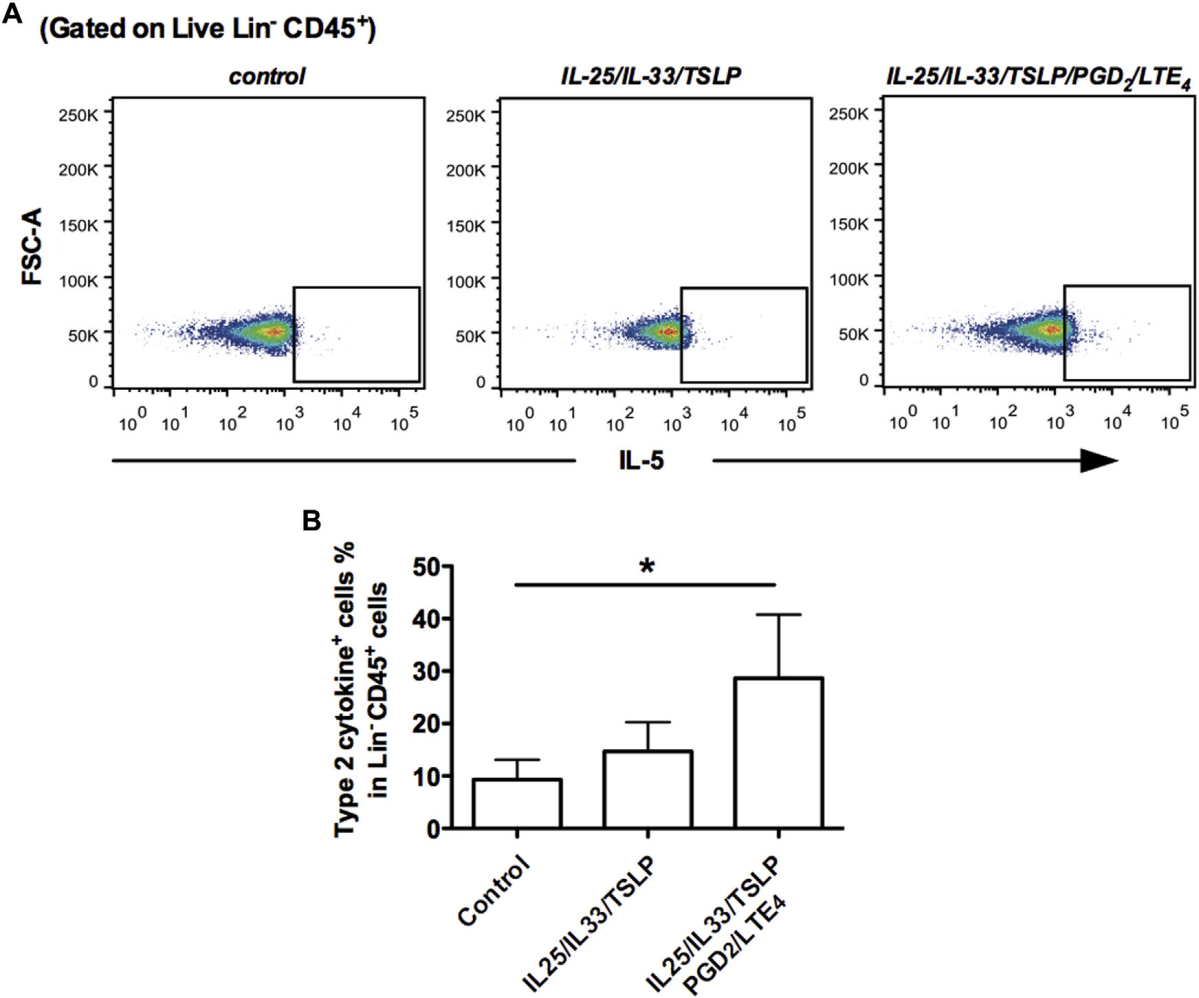
LTE_4_ and PGD_2_ enhanced the effects of epithelial cytokines on type 2 cytokine production by ILC2s *ex vivo*. **A,** IL-5^+^ ILC2s detected in fresh PBMCs after stimulation with IL-33 (50 ng/mL), IL-25 (50 ng/mL), and TSLP (50 ng/mL) or a combination of these epithelial cytokines with LTE_4_ (50 nmol/L) and PGD_2_ (100 nmol/L) by using intracellular staining. **B,** Proportion of type 2 cytokine (IL-5 and IL-13)–positive cells in gated Lin^−^CD45^+^ cells after indicated treatments. *P < .05. Data in Fig E5, *A*, are representative of 3 independent experiments (Fig E5, *B*, n = 3).

Next, we measured the synergistic effect of LTE_4_ and epithelial cytokines. Treatment with IL-33, IL-25, and TSLP alone for 4 hours showed only very weak induction of cytokine production by ILC2s (Fig 5, *B*). However, addition of IL-33, IL-25, or TSLP to LTE_4_ significantly augmented cytokine production (IL-4, IL-5, IL-8, IL-13, GM-CSF, CSF1, and amphiregulin). The greatest enhancement was seen when IL-25, IL-33, and TSLP were used in combination with LTE_4_. Intracellular staining for type 2 cytokines in ILC2s *ex vivo* also confirmed this enhancing effect (see Fig E5 in this article's Online Repository at www.jacionline.org). Montelukast abrogated the effect of LTE_4_.

**Fig 6:**
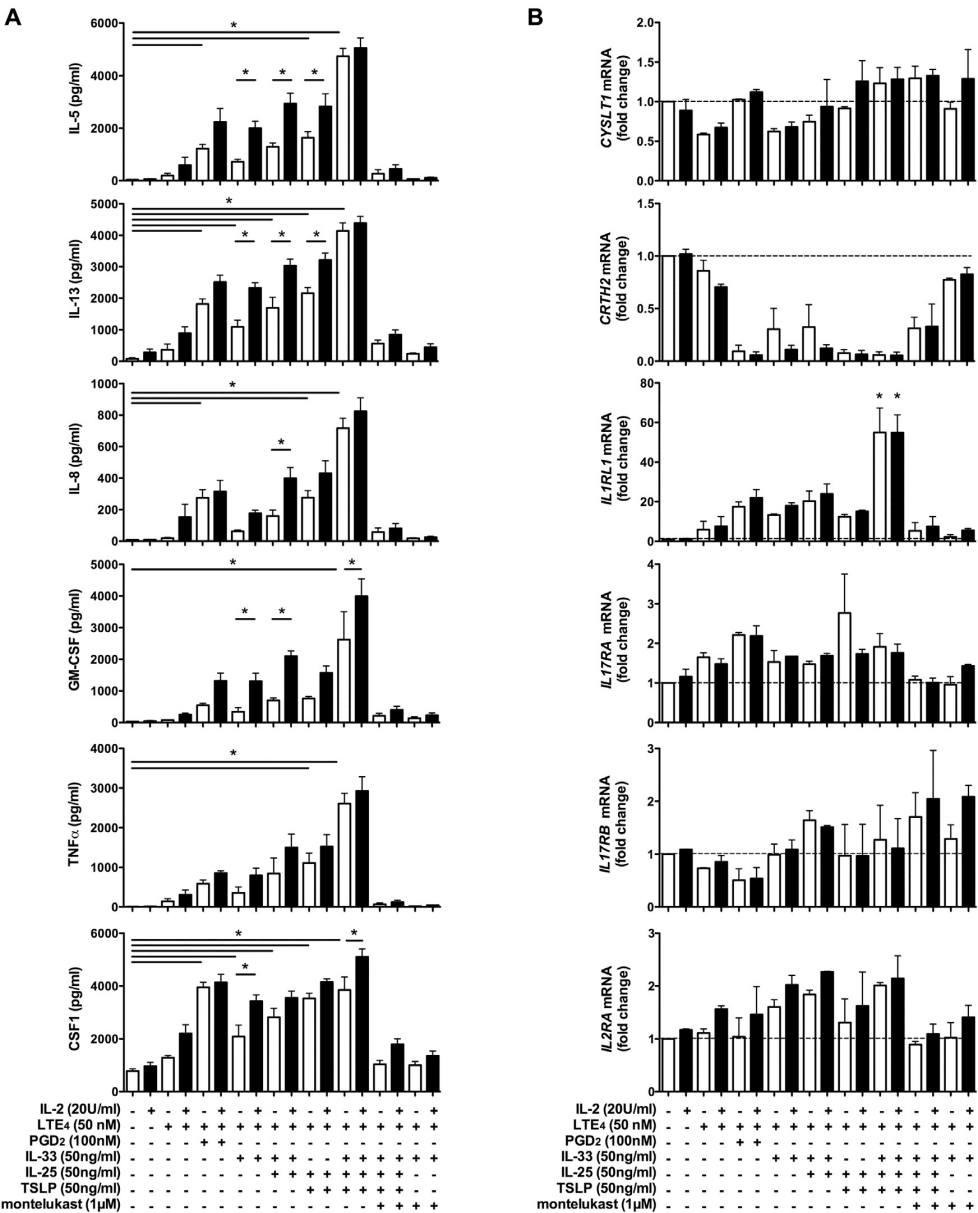
Effect of IL-2 and other stimulators on cytokine and receptor expression in cultured ILC2s. Cytokine concentrations in supernatants **(A)** or mRNA levels of receptors in ILC2s **(B)** after treatment with combinations of LTE_4_, PGD_2_, IL-33, IL-25, and TSLP with or without montelukast in the presence *(black bars)*/absence *(white bars)* of IL-2 measured with the multiplex bead array (Fig 6, *A*) or quantitative RT-PCR (Fig 6, *B*). *P < .05 (n = 3-5).

**Fig E6:**
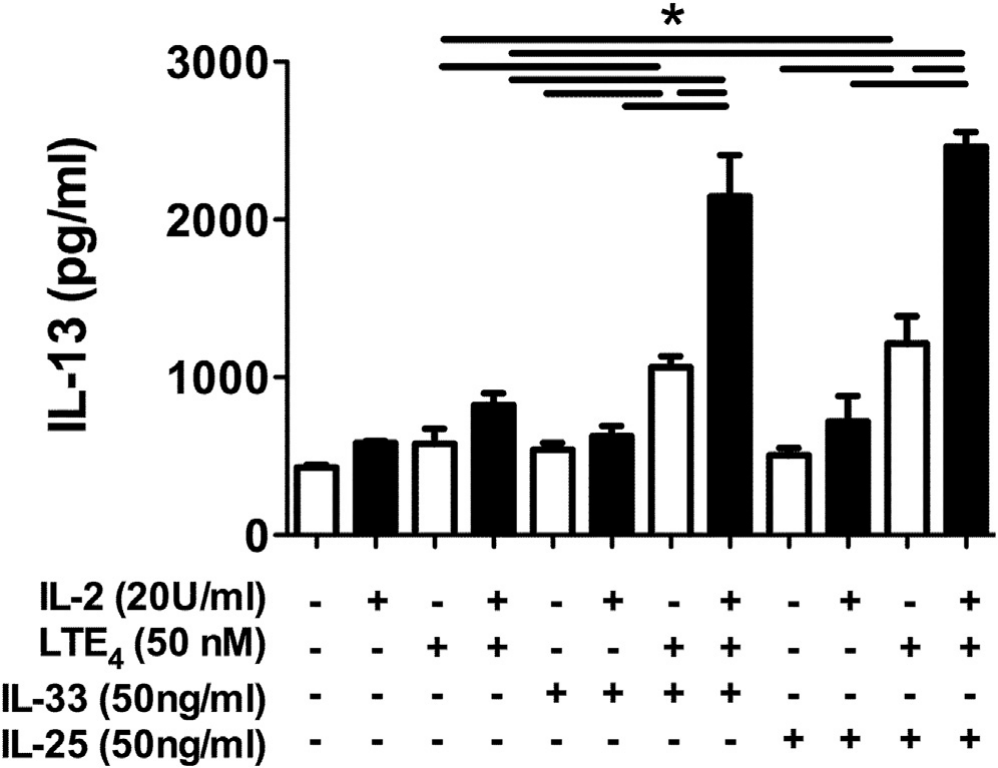
IL-2–enhanced cytokine production in ILC2s in response to LTE_4_, IL-33, IL-25, and their combinations. IL-13 concentrations in ILC2 culture after incubation with LTE_4_, IL-33, or IL-25 or their combinations in the presence *(black bars)* or absence *(white bars)* of IL-2 were measured with the multiplex bead array. *P < .05 (n = 3).

**Fig E7:**
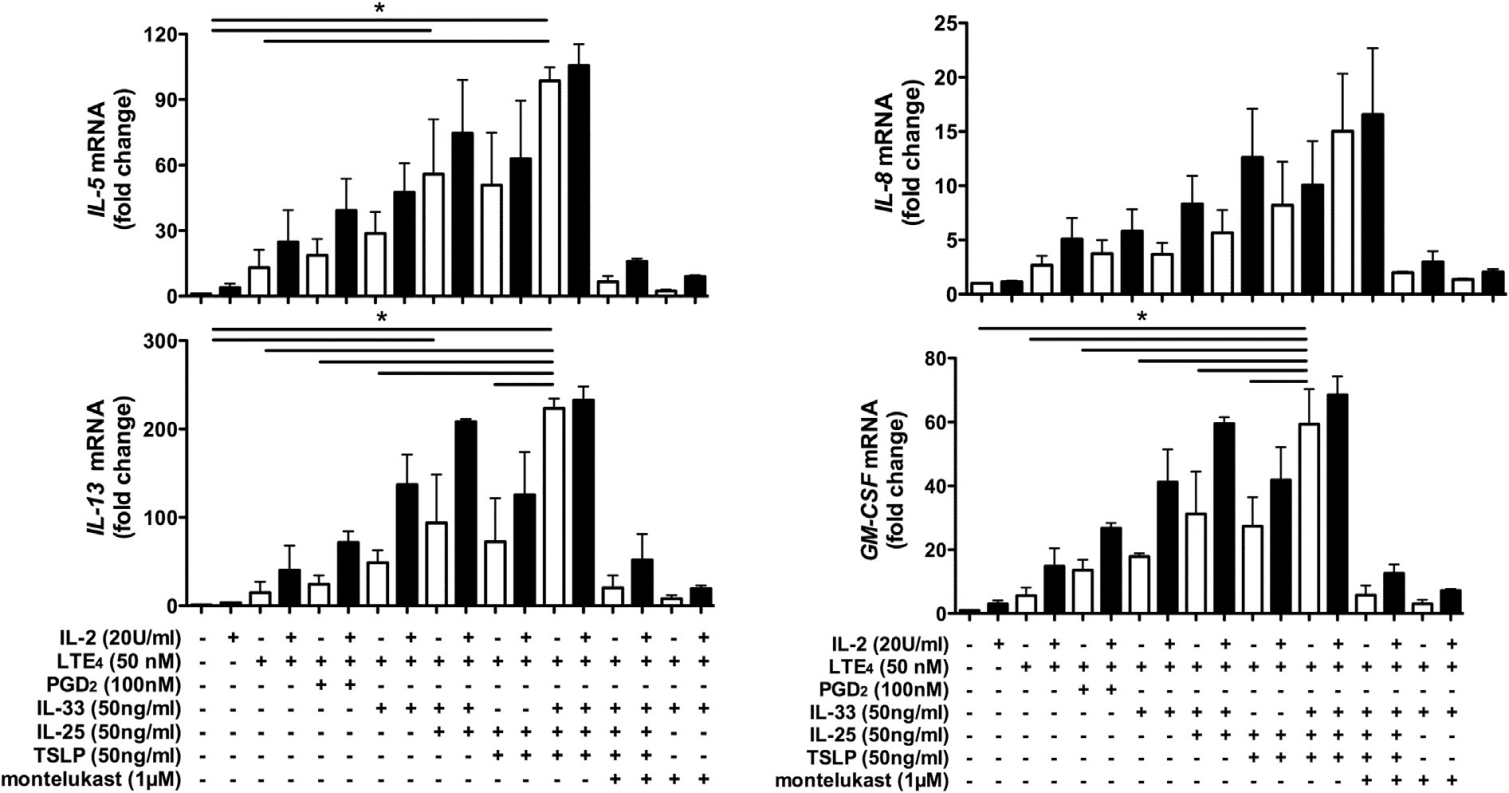
Effect of combination of IL-2 and other stimulators on gene expression of cytokines in ILC2 cells. mRNA levels of cytokines in cultured ILC2s after treatment with different combinations of LTE_4_, PGD_2_, IL-33, IL-25, and TSLP with or without montelukast in the presence *(black bars)* or absence *(white bars)* of IL-2 for 4 hours were measured with quantitative RT-PCR. *P < .05 (n = 3).

IL-2 is a critical cofactor in regulating ILC2 function in patients with lung inflammation.^28^ To understand the role of IL-2 in human ILC2 cytokine production, we compared cell treatments in the presence or absence of IL-2 (Fig 6). IL-2 alone had no detectable effect on the production of most cytokines. However, it slightly enhanced the cytokine production induced by LTE_4_ and epithelial stimulators alone and significantly enhanced the cytokine production induced by the combination of LTE_4_ and other stimulators, including the combination of LTE_4_ with all 3 epithelial cytokines (IL-33, IL-25, and TSLP; Fig 6, *A*, and see Fig E6 in this article's Online Repository at www.jacionline.org). Inhibition of CysLT_1_ abolished the synergistic enhancement. The effect of IL-2 was further confirmed at the level of gene expression (see Fig E7 in this article's Online Repository at www.jacionline.org).

**Fig E8:**
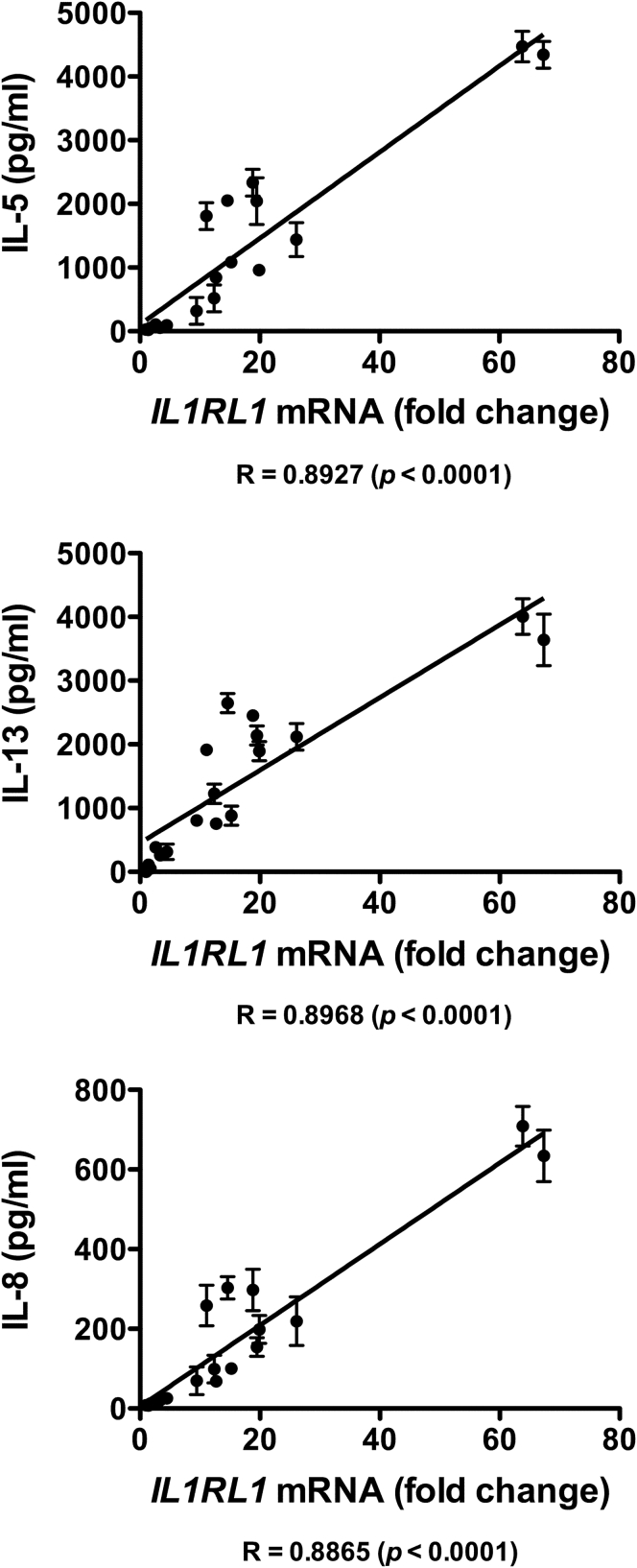
Relationship between *IL1RL1* gene regulation and cytokine productions in ILC2s after various treatments was analyzed by using Spearman analysis.

To further investigate the potential mechanism involved in the synergistic enhancement of ILC2 activation by the combination of LTE_4_ with other stimulators, we examined the mRNA levels of the relevant receptors *CYSLT1*, *PTGDR2*, *IL1RL1*, *IL17RA*, *IL17RB*, and *IL2RA* after treatment (Fig 6, *B*). Although *PTGDR2* (CRTH2) was strongly downregulated and *CYSLT1* and *IL17RB* were partially downregulated by the combined stimulations, *IL17RA*, *IL2RA*, and *IL1RL1* were upregulated. Upregulation of *IL1RL1* was particularly strong and showed strong correlation with cell activation detected based on cytokine production (see Fig E8 in this article's Online Repository at www.jacionline.org).

### Endogenous cysLTs from activated human mast cells stimulate ILC2s

**Fig 7:**
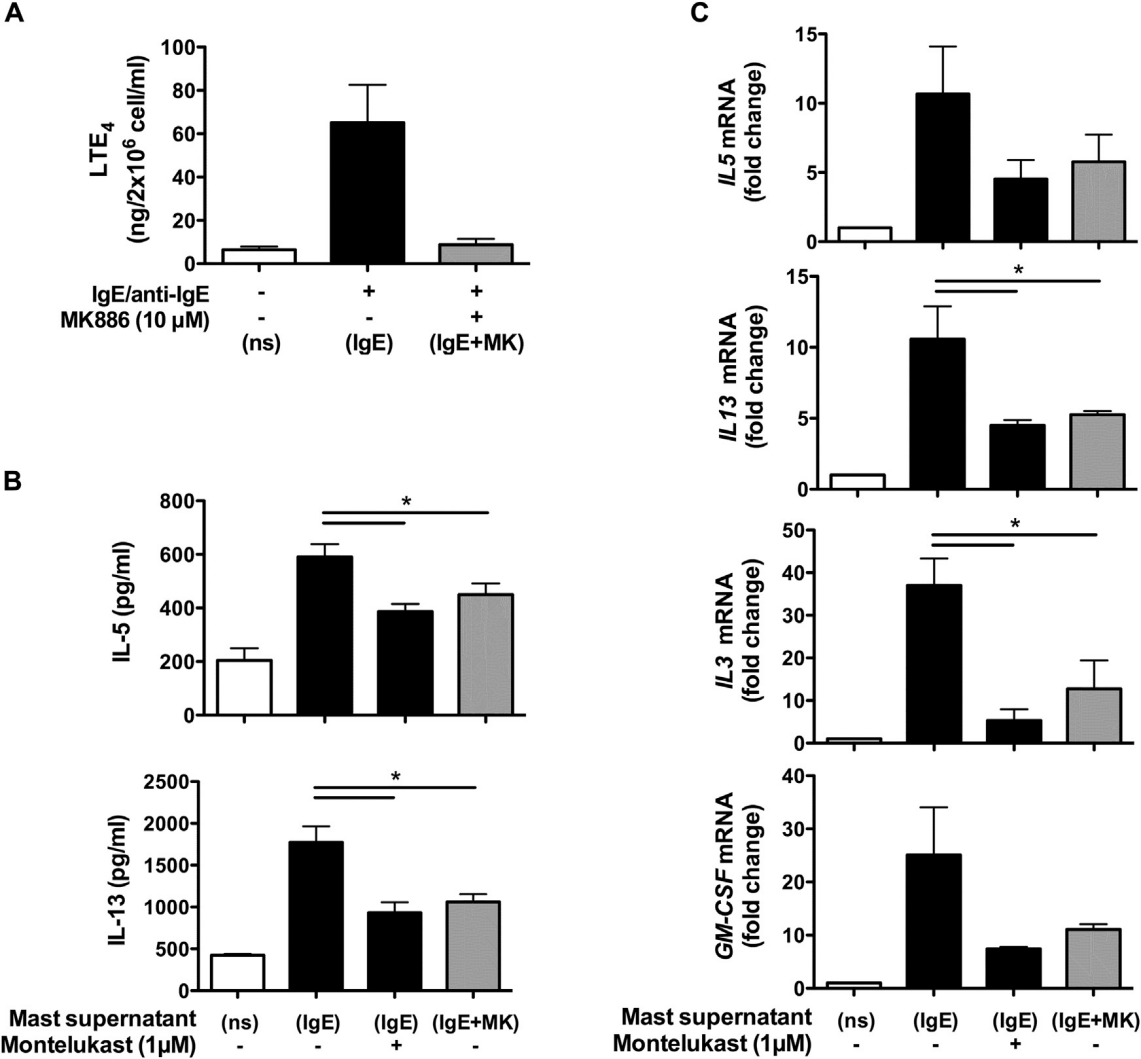
Effect of endogenous cysLTs on cytokine production in cultured ILC2s. **A,** Levels of LTE_4_ in supernatants of mast cells treated with medium (*white bar*, *ns*) or IgE/anti-IgE with (*gray bar*, *IgE+MK)* or without (*black bar*, *IgE*) MK886. **B** and **C,** Protein (Fig 7, *B*) and mRNA (Fig 7, *C*) levels of cytokines of ILC2s after incubation with supernatants with or without montelukast for 4 hours. *P < .05 (n = 4).

CysLTs are major lipid mediators produced by mast cells.^29^ The effect of endogenously synthesized cysLTs from activated human mast cells on ILC2s was examined to confirm the role of cysLTs in ILC2 biology under physiologic conditions (Fig 7). Only low levels of LTE_4_ (approximately 6 ng/2 × 10^6^ cells/mL) were detectable in supernatants from resting mast cells (Fig 7, *A*). Activation with IgE followed by anti-IgE antibody cross-linking of mast cells produced high LTE_4_ levels (approximatley 86 ng/2 × 10^6^ cells/mL). Cotreatment of IgE/anti-IgE–activated mast cells with MK886 (10 μmol/L), an inhibitor of 5-lipoxygease–activating protein, during the period of anti-IgE stimulation abolished LTE_4_ production (approximately 7.9 ng/2 × 10^6^ cells/mL). Using these supernatants to treat ILC2s revealed that the supernatant from IgE/anti-IgE–activated mast cells but not that of resting mast cells induced production of IL-5 and IL-13 (Fig 7, *B*). The type 2 cytokine production induced by the mast cell supernatant was partially but significantly inhibited by montelukast. Blocking cysLT synthesis in mast cells with MK866 also partially reduced the capacity of the supernatant to induce cytokine production by ILC2s. A similar effect was observed at the mRNA level (Fig 7, C).

## Discussion

Activation of ILC2s leads to production of type 2 cytokines, and therefore they represent a potential source of the dysregulated type 2 cytokine production seen in patients with eosinophilic inflammatory conditions, such as asthma and atopic dermatitis.^1^ This group of cells is found in the blood, spleen, intestines, liver, skin, fat-associated lymphoid clusters, and lymph nodes and is enriched in inflamed skin, nasal polyps, and lungs (data unpublished).^1^, ^2^, ^4^ Several previous studies have identified the innate epithelial cytokines IL-33, IL-25, and TSLP and the lipid mediator PGD_2_ as important stimulators for ILC2s.^5^, ^7^ Although cysLTs have been investigated in mice,^8^ in the current study we examined the previously unrecognized roles of cysLTs in human ILC2 activation. They promoted cytokine production, induced cell migration, and reduced apoptosis by ILC2s. These effects were also seen in response to endogenous cysLTs from human mast cells. LTE_4_ showed the highest efficacy among cysLTs in ILC2 biology, and the effects were blocked by the CysLT_1_ antagonist montelukast. The activation of ILC2s was significantly amplified when treated with cysLTs combined with PGD_2_ and epithelial cytokines, particularly in the presence of IL-2, suggesting an important proinflammatory role of cysLTs in ILC2-mediated immune responses in the presence of other ILC2 stimulators.

LTs are potent proinflammatory lipids predominantly derived from activated mast cells and basophils.^29^ Their biological functions are mediated through binding to a group of G protein–coupled receptors: CysLT_1_ and CysLT_2_.^9^, ^10^ Because both receptors have only low affinity for LTE_4_ based on ligand-binding assays,^16^, ^17^ the potent activity of LTE_4_ in human ILC2s suggested to us a potential CysLT_1_/CysLT_2_-independent mechanism. P2Y_12_ and GPR99 have been proposed as receptors with a preference for LTE_4_,^18^, ^19^ but we found that neither is expressed in ILC2s. A recent report demonstrated that CysLT_1_ is critically important for responsiveness to LTE_4_ within certain types of human cells.^30^ This could be the case for human ILC2s because (1) human ILC2s express high levels of CysLT_1_, (2) LTE_4_ showed higher efficacy than LTD_4_ in human ILC2s, and (3) the activity of LTE_4_ in human ILC2s was completely inhibited by montelukast. This phenomenon has also been observed previously in human T_H_2 cells.^14^, ^15^ However, we still cannot rule out the possibility of involvement of another unknown LTE_4_ receptor. If such a putative receptor exists, our data suggest that it should be responsive to montelukast. Mouse ILC2s also express CysLT_1_.^8^ However, in contrast to the findings in human ILC2s, the effect of LTE_4_ in mice seems weaker than that of LTD_4_. Furthermore, the IL-5–producing ILC2s induced by LTE_4_ administered *in vivo* cannot be inhibited by montelukast in mouse models (although this interaction was not dissected *in vitro*). These between-species differences in the effects of LTE_4_ on ILC2s might be consistent with involvement of a novel LTE_4_ receptor.

The level of CysLT_1_ in human ILC2s is upregulated in cells from patients with atopic dermatitis, suggesting a potential pathogenic role of cysLTs/CysLT_1_ in patients with the disease. Several studies have shown an improvement of atopic dermatitis after treatment with CysLT_1_ antagonists.^31^, ^32^, ^33^ Furthermore, a significantly higher concentration of LTE_4_ was detected in urine of patients with atopic dermatitis or asthma and in sputum from patients with asthma compared with healthy volunteers.^34^, ^35^, ^36^ Collectively, these findings suggest that LTE_4_-mediated ILC2 activation could be a critical contributor to allergic inflammation. This role might be particularly important in patients with aspirin-exacerbated asthma, in whom LTE_4_ overproduction and a clinical response to cysLT blockade are particularly obvious.^37^

It has been well established that epithelial cytokines (IL-33, IL-25, and TSLP) are important stimulators for ILC2 responses, and the lipid mediator PGD_2_ is also a strong regulator of ILC2s in type 2 immunity.^2^, ^7^, ^38^ Here we have shown that cysLTs have multiple proinflammatory effects on ILC2 migration, apoptosis, and cytokine production. They not only elicited production of type 2 cytokines but also other proinflammatory cytokines, including IL-8, GM-CSF, CSF1, TNF-α, and amphiregulin. These cytokines could, orchestrating with type 2 cytokines, contribute to eosinophilic (GM-CSF and TNF-α), neutrophilic (IL-8, GM-CSF, and TNF-α), and tissue-remodeling (CSF1, TNF-α, and amphiregulin) effects during allergic inflammations.^3^, ^39^, ^40^, ^41^ Such reactions can be confirmed in other types of cell systems inhibited by montelukast.^40^ The efficacies of cysLTs were significantly higher than those of innate cytokines (IL-33, IL-25, and TSLP) but lower than that of PGD_2_ in human ILC2s in *in vitro* assays when using them individually, indicating the important role of cysLTs in human ILC2s. In *in vitro* studies the ILC2 response to the lipid mediators is much faster (ie, hours) than that to the epithelial cytokines (ie, days).^2^, ^4^, ^7^ However, the speed of these responses under physiologic conditions will also depend on the timing of enrichment of these stimulators at inflammatory sites. To confirm the role of cysLTs under arguably more physiologic conditions, we examined the effect on ILC2s of endogenously synthesized cysLTs from human mast cells. The ILC2 response to the mast cell supernatant was similar to that seen with exogenously synthesized cysLTs. The only difference was that the response to the mast cell supernatant could not be completely blocked by montelukast or by inhibition of cysLT synthesis. This could be caused by the presence of other active mediators released from activated mast cells, such as PGD_2_.^29^ Thus cysLTs only partially deliver the stimulating signal from activated mast cells to ILC2s. CysLTs can also be generated by other cells of the innate immune system, such as basophils, eosinophils, and alveolar macrophages, after exposure to allergens, proinflammatory cytokines, and other stimuli during allergic inflammation and also transcellularly by platelet-adherent leukocytes in patients with aspirin-exacerbated asthma.^9^, ^29^, ^37^ Therefore cysLTs can contribute to IgE-independent innate responses of ILC2s. Given the association of tissue mast cells, basophils, and eosinophils with allergic dermatitis and asthma, the inhibition of cysLT-mediated ILC2 activation might provide a therapeutic opportunity for these diseases potentially when combined with other approaches.

Although cysLTs alone can activate ILC2s, the more pronounced effect was observed when they were used in combination with another lipid mediator (PGD_2_) or epithelial cytokines (IL-33, IL-25, and TSLP). We suggest this effect is more representative of *in vivo* conditions in which, after allergen encounter, LTs and prostaglandins are simultaneously secreted by mast cells in parallel with IL-33, IL-25, and TSLP production by a compromised epithelium. Synergistic enhancement between LTE_4_ and PGD_2_ was also observed in human T_H_2 cells.^14^, ^15^ Cross-enhancement of the effects of IL-25, IL-33, and TSLP has been reported in nasal epithelial and T_H_2 cells.^42^, ^43^ However, the effect of cysLTs on the activity of epithelial cytokines is unknown. Here, for the first time, we reported that LTs markedly enhanced the effect of epithelial cytokines on ILC2 cytokine production. Our data demonstrated that the receptors for IL-33 and IL-25 were upregulated by the combination treatment and therefore might contribute to the enhanced effects. In keeping with this, we found a positive correlation between the degree of upregulation of *IL1RL1* and enhancement of cytokine production under the same treatment. Further investigation is required to fully understand the mechanisms underlying this synergistic reaction.

Another interesting observation is the promoting role of IL-2 on the ILC2 cytokine production in response to the combination of LTE_4_ and epithelial cytokines. IL-2 greatly improved the stimulatory effect of LTE_4_ in combination with IL-33, IL-25, and TSLP, whereas the effect of IL-2 on individual stimulator or on the combination of LTE_4_ and PGD_2_ was minimal. *In vitro*, IL-2 is required in the culture for ILC2 growth and proliferation. *In vivo*, it has been reported that IL-2 is a cofactor in regulating ILC2 function in pulmonary inflammation and coordinating type 2 cytokine expression in mouse models.^28^, ^44^ Increased levels of IL-2 were detected in the lungs of asthmatic patients, and inhaled IL-2 therapy has been shown to induce asthma-like airway inflammation.^45^ Our data suggest a critical promoting effect of IL-2 on LTE_4_ and epithelial cytokine-induced activation of human ILC2s, which might be relevant to the pathogenesis of asthma and atopic dermatitis.

In summary, the present investigation highlights the multifaceted role of cysLTs, particularly LTE_4_, and their receptors in the activation of human ILC2s. They initiate production of type 2 and other proinflammatory cytokines, induce cell migration, and suppress cell apoptosis. Most importantly, they significantly enhanced the ILC2 response to other established ILC2 activators. The effects of cysLTs can be blocked by the CysLT_1_ inhibitor montelukast. Considering that increased levels of cysLTs (LTE_4_) and CysLT_1_ are associated with allergic diseases, such as atopic dermatitis and asthma, the findings in this study support the view that LTs play a pivotal role in ILC2-mediated allergic inflammation under physiologic conditions and represent an important drug target for related disorders, likely as part of therapeutic combinations.

**Key messages**

• Human ILC2s express functional CysLT_1_, levels of which are increased in patients with atopic dermatitis.
• CysLTs are important activators of human ILC2s, and LTE_4_ is the most potent among the cysLTs.
• LTE_4_ synergistically enhances the effect of epithelial cytokines in human ILC2s through upregulation of IL-33/IL-25 receptors.

## Figures and Tables

**Table E1:** Primers and probes used for quantitative RT-PCR

Gene	Primer	Probe no.
*IL3*	5′-TTGCCTTTGCTGGACTTCA-3′ 5′-CTGTTGAATGCCTCCAGGTT-3′	60
*IL5*	5′-GGTTTGTTGCAGCCAAAGAT-3′ 5′-TCTTGGCCCTCATTCTCACT-3′	25
*IL8*	5′-AGACAGCAGAGCACACAAGC-3′ 5′-ATGGTTCCTTCCGGTGGT-3′	72
*IL13*	5′-AGCCCTCAGGGAGCTCAT-3′ 5′-CTCCATACCATGCTGCCATT-3′	17
*GMCSF*	5′-TCTCAGAAATGTTTGACCTCCA-3′ 5′-GCCCTTGAGCTTGGTGAG-3′	1
*PTGDR2*	5′-CCTGTGCTCCCTCTGTGC-3′ 5′-TCTGGAGACGGCTCATCTG-3′	43
*CYSLT1*	5′-ACTCCAGTGCCAGAAAGAGG-3′ 5′-GCGGAAGTCATCAATAGTGTCA-3′	29
*CYSLT2*	5′-CTAGAGTCCTGTGGGCTGAAA-3′ 5′-GTAGGATCCAATGTGCTTTGC-3′	48
*GPR99*	5′-CAACCTGATTTTGACTGCAACT-3′ 5′-GGATAATCGTGGTATAGCAAAGTG-3′	16
*IL1RL1*	5′-TTGTCCTACCATTGACCTCTACAA-3′ 5′-GATCCTTGAAGAGCCTGACAA-3′	56
*IL2RA*	5′-ACGGGAAGACAAGGTGGAC-3′ 5′-TGCCTGAGGCTTCTCTTCA-3′	54
*IL17RA*	5′-CATCCTGCTCATCGTCTGC-3′ 5′-GCCATCGGTGTATTTGGTGT-3′	85
*IL17RB*	5′-GCCCTTCCATGTCTGTGAAT-3′ 5′-CCGGCCTTGACACACTTT-3′	64
*P2Y12*	5′-TTTGCCTAACATGATTCTGACC-3′ 5′-GGAAAGAGCATTTCTTCACATTCT-3′	27
*GAPDH*	5′-AGCCACATCGCTCAGACAC-3′ 5′-GCCCAATACGACCAAATCC-3′	60

*GAPDH*, Glyceraldehyde-3-phosphate dehydrogenase.

**Table E2:** Study subjects

	Patients with atopic dermatitis (n = 8)	Healthy control subjects (n = 4)
Female/male sex	5/3	3/1
Age (y)	30-88	28-46
SCORAD score	5-70	NA

*NA*, Not applicable.

## Methods

### Reagents

LTC_4_, LTD_4_, LTE_4_, and PGD_2_ were purchased from Enzo Life Science (Farmingdale, NY). Lymphoprep was purchased from Axis-Shield UK (Dundee, United Kingdom). The RNeasy Mini Kit and Omniscript Reverse Transcription Kit were supplied by Qiagen (Hilden, Germany). Real-time qPCR Master Mix and probes were from Roche (Mannheim, Germany). Primers were synthesized by Eurofins MWG Operon (Ebersberg, Germany). CD3 microbeads, the CD34 Microbead Kit, and anti-CRTH2 antibody were from Miltenyi Biotec (Bergisch Gladbach, Germany). Anti-human IL-13, CD3, CD19, CD45, and CD127 antibodies were from eBioscience (San Diego, Calif). Anti-human CD4, CD8, CD16, CD56, TCRαβ, TCRγδ, FcεRI, and IL-5 antibodies were from BioLegend (San Diego, Calif). Anti-human CD14 antibody was obtained from BD Biosciences. Anti-human CD11b and OXGR1 (GPR99) antibodies were from Abcam (Cambridge, United Kingdom). Anti-human CD11c antibody was from Life Technologies (Grand Island, NY). Anti-human CD123 antibody was from R&D Systems, and anti-human CysLTR1 antibody was from Novus Biologicals (Littleton, Colo). Recombinant human IL-2, IL-4, IL-25, IL-33, and TSLP were from PeproTech (Rocky Hill, NJ). Human IgE was purchased from Merck Millipore (Billerica, Mass). MK886 and montelukast were from Cayman Chemicals. Other chemicals were from Sigma-Aldrich (St Louis, Mo).

### Western blotting

Cell lysates from ILC2s, T_H_2 cells, and NIH3T3 cells were prepared with a lysis buffer (20 mmol/L Tris-HCl [pH 7.4], 250 mmol/L sucrose, 1 mmol/L EDTA, 1 mmol/L ethyleneglycol-bis-[b-aminoethy-lether]-N,N,N9,N9-tetraacetic acid, 10 mmol/L sodium glycerophosphate, 50 mmol/L sodium fluoride, 5 mmol/L sodium pyrophosphate, 0.1% 2-mercaptoethanol, protease inhibitor mixture, and 1% Triton X-100). Lysates were fractionated by using SDS-PAGE on NuPAGE Novex 12% Bis-Tris Gels precast gels (Invitrogen). Western blot assays for GPR99 (OXGR1) were carried out on polyvinylidene difluoride membrane (iBlot stacks; Invitrogen).

### Proliferation assay

Primary ILC2 cultures were washed with 0.1% BSA-PBS twice and resuspended in 500 μL of 0.1% BSA-PBS containing 5 μmol/L Cell Trace Violet (C34557, Life Technologies). After incubation at 37°C for 20 minutes, cells were washed twice with cold RPMI supplemented with 10% human serum. Labeled ILC2s were cultured in normal medium (RPMI 1640 containing 100 IU/mL IL-2, 10% heat-inactivated human serum, 2 mmol/L l-glutamine, 100 IU/mL penicillin, and 100 μg/mL streptomycin) without or with 100 nmol/L LTC_4_, LTD_4_, or LTE_4_. Levels of Cell Trace Violet in the cells were measured and compared at days 1 and 4 with CyAn flow cytometry.

### Intracellular cytokine staining

PBMCs were isolated from fresh blood and then stimulated with combinations of different cytokines and lipid mediators, as indicated, for 18 hours. Brefeldin A (3 μg/mL) was added during stimulation. Cells were washed twice with PBS. After cell-surface staining with lineage and CD45 antibodies and Live/Dead staining, cells were permeabilized with a Cytofix/Cytoperm kit (BD), followed by staining with IL-5 (clone TRFK5) or IL-13 (clone 85BRD) antibodies. Then the cells were washed and fixed with 0.5% formaldehyde. Samples were analyzed with an LSR Fortessa flow cytometer.

### Analysis of correlation

*R* values were calculated by using the Spearman test.
